# Advances in Target Detection and Tracking in Forward-Looking InfraRed (FLIR) Imagery

**DOI:** 10.3390/s141120297

**Published:** 2014-10-28

**Authors:** Andrea Sanna, Fabrizio Lamberti

**Affiliations:** Politecnico di Torino, Dipartimento di Automatica e Informatica, Corso Duca degli Abruzzi 24, Torino 10129, Italy; E-Mail: fabrizio.lamberti@polito.it

**Keywords:** forward-looking infrared imagery, infrared sensors, thermal, automatic target recognition, tracking, detection, classification, registration, features extraction, template matching and updating, activity recognition

## Abstract

Here we give context to the Special Issue on “Detection and Tracking of Targets in Forward-Looking InfraRed (FLIR) Imagery” in Sensors. We start with an introduction to the role of infrared images in today's vision-based applications, by outlining the specific challenges that characterize detection and tracking in FLIR images. We then illustrate why selected papers have been chosen to represent the domain of interest, by summarizing their main contributions to the state-of-the-art. Lastly, we sum up the main evidence found, and we underline some of the aspects that are worthy of further investigation in future research activities.

## 1. Introduction

Object detection and tracking represent key components of many vision-based applications, from surveillance to vehicle navigation, driver assistance, activity recognition, *etc.* Hence, a number of algorithms have been proposed in the literature to cope with issues deriving from variations in objects' appearance, occlusion conditions, sensor ego-motion, and so on. For years, research focused on the visible light spectrum, by developing effective solutions tailored to both single and multiple monocular or stereo cameras. Unfortunately, approaches based (solely) on visible light sensors proved to be unable to provide fully satisfactory results under poor visibility conditions.

Recently, technological advancements led to an important reduction in the production costs of infrared (IR) light sensors. Hence, infrared cameras, which had been for a long time confined to the military domain, started to be extensively used in the industrial and civil fields. Attention has been specifically devoted to long-wave infrared (LWIR) light in the range 8–12 nm and to forward looking infrared (FLIR) sensors, since they can see heat sources at night or through smoke, fog, haze, *etc.* In fact, in FLIR images, the intensity of an object mainly depends on its temperature and radiated heat and it is not influenced by light conditions and object surface features.

The IR spectrum was considered not only to deal with specific detection and tracking issues arising in particular visibility conditions, but also to integrate solutions originally working solely with visible light. Hence, an approach that is frequently pursued consists in fusing information coming from the two sensor categories, thus maximizing the positive effect of their strong points while limiting at the same time the impact of their constraints. For example, when detecting and/or tracking pedestrians in visible light imagery, clothes color may be used to resolve possible occlusions that may occur, e.g., in crowded environments. At night, an IR sensor would help to deal with the lack of color information. However, pedestrian signatures would become very similar and, in case of overlap, it would be very difficult (if not impossible) to separate them.

Detection and tracking in FLIR images are complicated by other important factors. In fact, real-life images captured by surveillance cameras, Unmanned Aerial Vehicles (UAVs) and other stationary and non-stationary sensors are generally characterized by limited resolution, poor contrast and low signal-to-noise ratio. Nonetheless, important synergies could be observed as well, as there exist challenges that are almost shared between the infrared and visible domain. For instance, still making reference to the example above about pedestrian detection and tracking, both the visible light and IR techniques have to cope with the difficulties associated with a signature that is continuously changing due to the non-rigid nature of the human body, whose trajectories are hard to predict because of the intrinsic complexity of human walking motion and social behaviors, *etc.*

In this Special Issue we tried to summarize some of the main trends, which can be identified in today's strategies for target detection and tracking in FLIR imagery, with the aim to stress the growing attention that infrared spectrum-based algorithms, and related sensors, are receiving today in a rather wide number of scenarios.

## 2. Content

A total of 11 articles were finally published in this Special Issue, covering a variety of topics related to the analyzed domain. A first way to introduce them could be to consider the heterogeneous application fields tackled, which range from dim target detection from ground-based Earth Observation (EO) or sea vessel-mounted sensors [1,2], to the automatic tracking of humans [3–6], animals [7] and military vehicle [8,9] targets to semantic annotation of video sequences [10]. Another interesting point of view that could be adopted relates to the specific issues of detection and tracking in FLIR imagery that are actually addressed by the reported techniques: image registration [11], target representation and recognition [3,7–9], occlusion handling [4,6], multi-target association [10], clutter removal [1,2] and latency reduction [5]. Lastly, an interesting perspective is obtained by considering whether the various works propose new algorithms specifically designed to cope with the peculiarities of FLIR imagery [2,3,5–7], adapt or combine well-known techniques (like sparse coding, particle filtering, *etc.*) often used with visible light images to suit the requirements of the infrared spectrum [1,4,8], or simply study how existing alternatives would perform in the considered domain with the aim to stimulate further research [9,11]. In the following, we will review the various works, by summarizing their main contributions to the state-of-the-art.

In particular, Li *et al.* [1] report on the design of a new sparse representation-based algorithm for dim target detection tailored to infrared images generated by ground-based EO sensors. In the considered scenario, targets are generally very small (only a few pixels) and are usually submerged in noise and clutter. They move from considering that, when working on non-structured targets, the adoption of an adaptive morphological component over-complete dictionary (or, dictionary, for brevity in the following) could improve the sparsity of the representation coefficient vector over the use of a structural complete dictionary. Nonetheless, having atoms representing target and background mixed together, the former dictionary might still lack the level of sparsity required to properly detect target signals. Common approaches to address the above limitation consist in manually discriminating target signals to build a target(-only) dictionary (and in exploiting background clutter to train a background dictionary). However, these off-line operations prevent atoms in the discriminative dictionary from being capable of properly adapting to moving targets and changing background. Hence, the authors propose a technique for discriminating target atoms in an automatic way. In particular, they first build an adaptive morphological non-structural dictionary using K-singular value decomposition (K-SVD). Then, a target dictionary is discriminated online from a background dictionary by the criteria that target atoms could be decomposed more sparsely over a Gaussian dictionary than background ones. Experimental results showed that the proposed technique can achieve better performance compared to approaches based on Gabor and Gaussian structural dictionaries, by also strengthening the sparse feature difference between target and background than with dictionaries that are learnt online and classified in a manual way.

Kim and Lee [2] focus on sea-based infrared search and track, and propose a region-adaptive clutter rejection method for small targets detection capable to reduce the influence of false detections due to clouds, edge line of the horizon and sun-glint. The technique consists of a background processing and a target processing phases. In the background processing phase, sensor pose information is used to perform an automatic segmentation of sky, horizon and sea regions, which are then separately dealt with in the target processing phases. In particular, a modified mean subtraction filter (MSF) is used on the horizon region to remove the clutter created by heterogeneity across the horizontal line (and the line itself), whereas a local background adaptive two-threshold framework is used to decide which pixels belong to target objects. The sky region is processed by an AdaBoost classifier that exploits various spatial features (standard deviation, 2nd order moment, size ratio, rotational size variation, *etc.*) to remove false detections from cloud clutter. Lastly, the sea region is processed by using a temporal consistency filter based on three-plot correlation. During experimental tests, the components of the devised method have been compared with conventional techniques, showing improved performance in terms of computational complexity and/or false detection capabilities with comparable or slightly degraded detection rates.

Fernández-Caballero *et al.* [3] present an interesting algorithm for robust extraction of pedestrian regions of interest (ROIs) from IR video sequences, based on image and motion-related information. The algorithm includes two preliminary steps, referred to as “thermal analysis” and “motion analysis”, which are carried out in parallel. In the former step, a set of candidate ROIs that are assumed to contain one or more pedestrians are extracted by applying adaptive thresholding and morphological operations. In the latter, image subtraction is performed over two consecutive frames, and so called “warm” pixels are identified as those whose difference between the two frames is over a given threshold; connected “warm” regions constitute another set of candidate ROIs. Results from the above preliminary analysis steps are processed by a “ROI fusion” stage, which is responsible for creating a newer set of ROIs by summing up or overlapping the candidate ones. A histogram-based filtering technique is then applied to intensity values in ROIs columns to check for the presence of multiple pedestrian groups. If a ROI is found to contain more than one pedestrian, sub-ROIs are extracted, each assumed to contain one pedestrian only. A final stage compares ROIs aspect ratio, standard deviation and size with reference values for the human body to confirm the actual presence of a pedestrian.

Li *et al.* [4] explore the relations (dependencies) between recognition and tracking, by proposing an automatic target recognition and tracking (ATR) framework based on sparse coding. Information coming from tracking the same object over consecutive frames is used to dynamically update the dictionary with target templates, thus enhancing recognition performance (“tracking-for-recognition”); at the same time, recognition of a given object provides information that is used to improve tracking of multiple objects, e.g., in case of frequent occlusions (“tracking-by-recognition”). Thus, they first use temporal redundancy deriving from knowing that the same object is represented (at different times) by different templates, by treating each template as an independent classifier and combining results from multiple classifiers together. Then, they implement a nonlocal dictionary update strategy that, based on recognition results, switches to a default set of templates when there is a significant probability that occlusion is occurring/has occurred. Experimental observations demonstrate that their joint tracking-and-recognition approach could significantly improve the accuracy and robustness of (pure) sparse-coding recognition and boost the performance of sparse coding-based tracking in case of occlusions.

Paravati and Esposito [5] consider the problem of ATR in FLIR imagery from the point of view of computational complexity and propose a mechanism for further improving the performance of high-speed tracking methods based on template matching by reducing the domain space using a relevance-based technique. In particular, they focus on a well-known tracking filter with an extremely small kernel based on intensity variation function and use a dynamic threshold on the correlation degree computed by the above function on consecutive frames to reduce the number of points to be processed. Tests carried out on sequences from different publicly available datasets showed that the proposed solution is capable of dramatically reducing the (already low) latency of the reference method by maintaining a robustness that is roughly comparable, at the cost of a rather high number of parameters that need to be experimentally defined.

Gade and Moeslund [6] focus on the problem of occlusions, and present an algorithm for tracking pedestrians in infrared images that exploits a novel technique to separate blobs containing partially-occluded pedestrians and reconstructs possibly split shapes corresponding to a single pedestrian. Tall blobs are split by analyzing convex hull convexity defects, whereas wide blobs are separated by considering their aspect ratios and perimeters. Erroneously split blobs are connected by using a rectangular mask whose size is supposed to be capable to contain a pedestrian and by computing the ratio of foreground pixels in that region. If the ratio is above a certain threshold, the overlap with other close rectangles is evaluated and used to determine whether the considered blobs came from the same shape. The above occlusion handling technique is integrated with a Kalman-based predictor and a continuous energy minimization strategy (CEM) into a multi-target tracking solution. Although the algorithm requires a number of parameters to be defined, promising experimental results carried out on sport videos confirmed the applicability of the proposed technique to the domain considered.

Christiansen *et al.* [7] propose an algorithm for automatically detecting and classifying animals in IR images to prevent wildlife mortality due to agricultural machinery. Detection is based on a novel thermal signature that is partly invariant to translation, rotation, scale and posture. At any frame, dynamic thresholding is exploited to detect possible ROIs. An iterative technique is then applied to extract all the possible contours of any given ROI (by removing at each iteration the extracted contour). ROI signature is represented as the profile defined by the mean intensity value of the contour at each iteration. Signature is normalized and approximated by discrete cosine transform (DCT) coefficients. Classification has been implemented by using k-Nearest Neighbors and incorporating temporal (tracking-based) information. Experimental results obtained with images captured from an elevated platform allowed the authors to make interesting observations that will eventually drive next efforts towards the integration of their technique in real-life monitoring environments, e.g., involving UAVs.

Gong *et al.* [8] propose an interesting algorithm for tracking, recognition and segmentation based on a shape-aware level set framework. The designed method supports robust ATR for different targets under arbitrary view angles. First, target shape is modeled by relying on a joint view-identity manifold (JVIM). JVIM is learned from 2D shapes generated from a set of 3D CAD models, and represents shape variability across different target types and views. By considering that different targets have different view manifolds but keep their identity independent of the view, the authors defined the JVIM so that it embeds one view-independent identity manifold and infinite identity-dependent view manifolds, which can then be interpolated to describe the targets. Then, recognition is implemented by implicit shape matching (not requiring any pre-processing or feature extraction step) through a shape-aware level set energy function (that additionally acts as a segmentation method). Finally, tracking is performed by introducing a (particle filter-based) dynamic motion model supporting robust sequential shape inference. Experimental tests carried out on a public dataset confirmed the advantages of the developed technique over explicit shape matching approaches.

Alam and Bhuiyan [9] provide a comprehensive overview of the characteristics of recently reported matched filter correlation techniques, which have been proved to be capable of providing quite interesting results (mainly in terms of computational complexity and robustness) when applied in aperture and laser radar imagery, but have not been widely exploited yet in the infrared domain. Then, they experimentally analyze which performances can be achieved by some such methods when used for automatically tracking targets in real-life FLIR image sequences. In particular, they focused on the (Extended) Maximum Average Correlation Height (E/MACH) and the (Polynomial) Distance Classifier Correlation Filter (P/DCCF) filters, which were exploited in the detection and classification steps, respectively. Preliminary results showed that matched filter correlation techniques could represent a valuable alternative for ATR in FLIR imagery, although ad-hoc strategies would be required to dynamically adapt the target model based on the particular characteristics of the image sequence being analyzed.

Hammoud *et al.* [10] present an innovative proof-of-concept solution for automatically annotating and registering image and text data produced in the context of aerial surveillance missions. Image data are first processed by a multiple ATR algorithm consisting of a frame-to-frame stabilization phase, which is aimed to register two consecutive images by dealing with possibly abrupt displacements, and a target detection and classification phase, which consists of a frame differencing step, a point-velocity pairs extraction step and a motion clustering step. A SVM classifier is used to distinguish people from vehicles and other objects. Variations of target attributes (like location, direction, speed) are broken down in semantic segments and are recorded in a graph-based representation. By following a similar approach, separate graphs are used to represent text information extracted from parsed chat messages. A multi-source graph association algorithm is used to link meaningful content in a chat message to the corresponding segment of a given tracked object. An unsupervised learning algorithm is then used on the above data to learn activity patterns, which could be exploited for query and visualization purposes in order to help the users quickly search, browse and analyze mission data.

Finally, Ricuarte *et al.* [11] study the behavior of well known computer vision techniques designed for visible light images when applied in another domain. In particular, they focus on classical feature point descriptors like SIFT, SURF, ORB, *etc.*, and analyze how they perform in the infrared spectrum, where images have less features than in the visible light domain and a lower number of matches is expected to be found. Experimental observations allowed them to identify the algorithms presenting the lowest sensitivity to rotations, scale changes as well as blur and noise effects under the considered conditions.

## 3. Conclusions

The richness and diverseness of the papers submitted to this Special Issue confirm the importance that FLIR and, more in general, infrared imagery, are assuming in an ever wider range of vision-based applications. In fact, results reported for the various techniques in their respective domains show that detection and tracking can already achieve, in the considered context, significant performance in terms of accuracy, robustness, speed, *etc.* Nonetheless, due to the particular challenges that are faced in the infrared spectrum, a trade-off has to found between algorithm efficiency and effectiveness, on the one side, and flexibility, on the other size. In particular, it can be observed that many of the approaches that have been presented need a number of parameters to be experimentally configured in order to provide the expected results. Hence, as stated by the authors, future work will have to be devoted to improve the ability of the various solutions to adapt to the specific features of the sequences under analysis. Moreover, an aspect that is quite common is the lack for a comprehensive comparison with alternative techniques on a reference ground. As underlined by the authors, this is often due to the availability of common datasets and public implementations, which is still quite limited. Hence, in the future, much more attention will have to be devoted to the topic of reproducibility of results.
